# Health Education for Musicians

**DOI:** 10.3389/fpsyg.2018.01137

**Published:** 2018-07-16

**Authors:** Raluca Matei, Stephen Broad, Juliet Goldbart, Jane Ginsborg

**Affiliations:** ^1^Centre for Music Performance Research, Royal Northern College of Music, Manchester, United Kingdom; ^2^Research and Knowledge Exchange, Royal Conservatoire of Scotland, Glasgow, United Kingdom; ^3^Faculty of Health, Psychology and Social Care, Manchester Metropolitan University, Manchester, United Kingdom

**Keywords:** health education, health promotion, musicians, course design, course evaluation

## Abstract

**Context and aims:** Many musicians suffer for their art, and health is often compromised during training. The Health Promotion in Schools of Music (HPSM) project has recommended that health education should be included in core curricula, although few such courses have been evaluated to date. The aim of the study was to design, implement and evaluate a compulsory health education course at a UK conservatoire of music.

**Methods:** The course design was informed by a critical appraisal of the literature on musicians' health problems and their management, existing health education courses for musicians, and the HPSM recommendations. It was delivered by a team of appropriately-qualified tutors over 5 months to 104 first-year undergraduate students, and evaluated by means of questionnaires at the beginning and end of the course. Thirty-three students who had been in their first year the year before the course was introduced served as a control group, completing the questionnaire on one occasion only. Items concerned: hearing and use of hearing protection; primary outcomes including perceived knowledge and importance of the topics taught on the course; and secondary outcomes including physical and psychological health and health-promoting behaviors. The content of the essays written by the first-year students as part of their course assessment served as a guide to the topics they found most interesting and relevant.

**Results:** Comparatively few respondents reported using hearing protection when practicing alone, although there was some evidence of hearing loss, tinnitus, and hyperacusis. Perceived knowledge of the topics on the course, and awareness of the risks to health associated with performing music, increased, as did self-efficacy; otherwise, there were negative effects on secondary outcomes, and few differences between the intervention and control groups. The topics most frequently covered in students' essays were managing music performance anxiety, and life skills and behavior change techniques.

**Conclusion:** There is considerable scope for improving music students' physical and psychological health and health-related behaviors through health education, and persuading senior managers, educators and students themselves that health education can contribute to performance enhancement.

## Introduction

Although many classical musicians derive positive emotions from their music making and find their profession meaningful (Ascenso et al., 2017), they can also suffer for their art. The largest survey to date was conducted by Fishbein et al. (1988). Seventy-six percent of 2,212 players from 47 American orchestras reported a medical problem severe enough to impair performance. The most prevalent were musculoskeletal, affecting the shoulder (20%), neck (22%), and back (16%). They also reported acute anxiety (13%), depression (17%), and sleep disturbances (14%). More recent research shows that musicians experience hearing loss (O'Brien et al., 2014), visual problems (Beckers et al., 2016), and eating disorders (Kapsetaki and Easmon, 2017). In addition, there is a higher prevalence of insomnia and psychological distress among musicians than in the general population, and they may be more likely to use psychotherapy and psychotropic drugs such as sedatives, antidepressants, hypnotics and/or medication for attention deficit hyperactivity disorder (ADHD) (Vaag et al., 2016a,b,c).

When compared with students in a variety of health-related disciplines, music students reported suffering from more varied and more severe symptoms (Spahn et al., 2004; Ginsborg et al., 2009; Panebianco-Warrens et al., 2015). When compared with age-matched members of the general population, undergraduate and postgraduate students from 10 British conservatoires showed higher levels of wellbeing and lower levels of fatigue. However, they also scored lower on measures of health responsibility, stress management, sleep quality, self-rated health, and use of coping skills (Araujo et al., 2017).

### Performance-related musculoskeletal disorders

Performance-related musculoskeletal disorders (PRMDs) have been defined as symptoms that have a negative impact on the ability to play (Zaza and Farewell, 1997). Studies of the prevalence of PRMDs in musicians playing a wide variety of instruments have now been conducted in many European countries, the USA, Brazil and Asia. Prevalence ranges between 26 and 93% (Zaza, 1998; Bragge et al., 2006; Ranelli et al., 2008; Leaver et al., 2011; Paarup et al., 2011; de Souza Moraes and Antunes, 2012; Ackermann et al., 2014; Arnason et al., 2014; Silva et al., 2015; Steinmetz et al., 2015; Kok et al., 2016; Lonsdale and Kuan Boon, 2016; Ciurana Moñino et al., 2017; Stanek et al., 2017). Kok et al. (2013) suggest that music students might experience musculoskeletal symptoms considerably more often than medical students, especially in the upper body and upper extremity.

A wide range of risk factors is associated with PRMDs. These can be psychosocial, such as anxiety, stress, perfectionism, and/or physical, including playing conditions such as temperature or length of rehearsals and performance and insufficient break periods, past injury, awkward posture and instrumental technique, poor fitness level and suboptimal injury management (Zaza and Farewell, 1997; Bragge et al., 2006; Kaufman-Cohen and Ratzon, 2011; Leaver et al., 2011; Chan and Ackermann, 2014; Steinmetz et al., 2015; Kochem and Silva, 2017).

### Music performance anxiety (MPA)

Like other forms of performance anxiety, including those related to test-taking, public speaking, and writing, MPA is a complex phenomenon with multiple causes encompassing genetics and environmental factors, but also personal experience, thoughts, emotions, and behaviors (Kenny, 2011). Although a certain level of arousal can facilitate optimal performance and a certain degree of anxiety is part of the normal bodily response to perceived threat, the differences between facilitating and debilitating anxiety, and between anxiety as a normal response to a relevant situation or context and anxiety as a mental disorder, may not be made explicit often enough (Miller and Chesky, 2004; Osborne et al., 2014; Kenny et al., 2016). Music students may struggle with anxiety more than experienced players (Kenny et al., 2012).

### Hearing loss

Musicians are regularly exposed to sound volume levels that exceed the recommended limit of 85 dB(A), which can produce noise-induced hearing loss (NIHL) and/or cause other disorders and symptoms such as tinnitus, hyperacusis, distortion, and diplacusis, some of which are incurable (Laitinen, 2005; Health Safety Executive, 2008; Santucci, 2009). Although sound exposure depends on variables such as instrument and repertoire played (Schmidt et al., 2011), and exposure time and environment, such as surrounding instruments and seating arrangements (Behar et al., 2006), musicians have an almost 4-fold higher hazard ratio (HR) for NIHL and 57% higher HR for tinnitus when compared to the general population (Schink et al., 2014). O'Brien et al. (2014) conducted a study of almost 600 musicians from eight Australian orchestras and found that 43% reported hearing loss. Although music students and professional musicians are recommended to use hearing protection, and they report receiving appropriate information about it, devices such as ear plugs may well be underused (Laitinen, 2005; Miller et al., 2007; Zander et al., 2008). However, in countries where legislation has raised awareness of potential hearing problems, usage has increased: for example, in Australia, 64% of the orchestral musicians surveyed by Ackermann et al. (2014) reported using hearing protection.

Efforts to educate orchestral musicians as to the effects of sound exposure have been made over the past two decades, most notably in the UK and Australia. In 2001 the Association of British Orchestras (ABO) published “A Sound Ear,” offering practical guidance and training for symphony orchestras (Wright-Rei et al., 2008). In 2011, the British Broadcasting Corporation (BBC) published a similar document in the form of a guide for musicians and a toolkit for managers (Hansford, 2011a,b,c).

### Health education and health promotion

Health promotion is “a process of enabling people to increase control over, and to improve their health” (Rootman et al., 2001, p. 13), thus empowering individuals. The Ottawa Charter for Health Promotion, based on the Report to the Canadian Ministry of Health (Lalonde, 1974) was adopted in 1986 (Chesky et al., 2006). It defines public health in relation to supportive settings: healthy working and living environments; health promotion as part of the daily activities of the setting; and links with the wider community (Dooris et al., 2010). These are also known as “settings for health” or “healthy settings,” that is, organizational structures in which health promotion may take various forms, such as schools, work sites, hospitals, villages, and cities.

One example of a healthy setting is the health-promoting school. Dooris et al. (2010) argue that the university context is particularly well-suited to promoting public health. The UK Healthy Universities Network^1^ was established in 2006 as a small and informal group, but has grown with support from the Higher Education Funding Council of England (HEFCE). The Network currently brings together 83 HEIs, 20 non-UK HEIs and 24 other stakeholder organizations, creates electronic tools, shares best practices and facilitates national projects. It also signed the Okanagan Charter for Health Promoting Universities and Colleges, an international framework for health promotion in higher education and post-secondary sector. The Charter uses a whole-system approach, taking advantage of every opportunity for health promotion: engaging all stakeholders (e.g., students, staff, administrators) in decision-making processes; facilitating interdisciplinary and cross-sector collaborations; promoting evidence-based policies, and practices; building strengths based on an informed understanding of contexts and local social landscapes (e.g., values, cultural diversity, etc.); and acting on the basis of the universal human right to health (Okanagan Charter, 2015). The Healthy Conservatoires Network^2^, modeled on the Healthy Universities Network, was established in 2015 as an outcome of the UK Arts and Humanities Research Council (AHRC)-funded Musical Impact project (see below).

Health promotion implies both increasing individuals' health literacy (via health education: building health-related knowledge and developing life skills to enhance capability, motivation, and self-efficacy) and trying to improve broader socio-economic, political and environmental living conditions by building supportive environments, encouraging community action, informing public health policies and ensuring that health services are oriented toward achieving population health outcomes. Life skills include stress management and emotional self-regulation, communication, and interpersonal skills, decision making, problem solving, critical thinking, and creative thinking (WHO, 1998). Given the complexity of health promotion, it should be distinguished from health education, which is more appropriately delivered through higher education curricula.

The US-based Health Promotion in Schools of Music (HPSM) project (2004–2006) resulted from a collaboration between the University of North Texas and the Performing Arts Medicine Association, and aimed to prevent musicians' playing-related occupational injuries. Their consensus-based recommendations aim to incorporate health-supportive environments and better educational programmes as part of professional music training, and include adopting a health promotion framework; developing and offering an undergraduate occupational health course for all music majors; educating students about hearing loss as part of ensemble-based instruction; and assisting students through active engagement with health care resources (Chesky et al., 2006).

Musical Impact was a 4-year AHRC-funded research project that brought together nine conservatoires in the UK to investigate and enhance the health and wellbeing of student and professional musicians (Musical Impact, 2013–2017) (Chesky et al., 2006). The project encompassed three strands: (1) Fit to Perform, investigating physical and mental performance fitness, as well as musicians' health-related attitudes and behaviors; (2) Making Music, exploring the physical and psychological demands of music making; and (3) Better Practice, focusing on interventions to promote health. The present study emerges from the Better Practice strand and investigates the effectiveness of a compulsory health education course, Health and Wellbeing for Musicians, for undergraduate music students.

### The present study

The course provided health education for first year undergraduate music students at a UK music conservatoire. The aims of the study were to (a) explore students' hearing and use of hearing protection; (b) design an evidence-based health education course; (c) assess the effects of the course on primary outcomes (perceived knowledge of course content and knowledge and awareness of potential risks to health) and secondary outcomes (including general health, health-related quality of life [HRQoL], health-promoting behaviors, self-efficacy, emotional state, perceived stress, frequency and severity of PRMDs, and perceived exertion); and (d) identify the topics most salient to students on the basis of the issues they chose to engage with in their course assessment.

## Materials and methods

### Health and wellbeing for musicians: course design

The course reflected all HPSM recommendations other than the adoption of a single health promotion framework. Its curriculum was informed by the findings of research on MPA and PRMDs; the findings of evaluations of other courses designed to improve musicians' health; theories and models deriving from health psychology (Taylor, 2012); discussions with the Acting Head of Undergraduate Studies at the institution where the first and fourth authors are based; and members of the Healthy Conservatoires Network.

Interventions designed to prevent and/or mitigate MPA were reviewed systematically by Burin and Osorio (2016), see also Matei and Ginsborg (2017), Spahn et al. (2016), Steyn et al. (2016), and Juncos et al. (2017). Interventions designed to prevent and/or mitigate PRMDs have been evaluated by Ackermann et al. (2002), Chan et al. (2014a,b), Kava et al. (2010), and Lee et al. (2012).

Few evaluations of health courses for musicians have been published, although they are delivered worldwide (Manchester, 2007a,b,c). Typical courses address mental and physical health, work satisfaction and coping, time management, wellbeing, and performance quality. They include information on general lifestyle, such as nutrition, physical fitness, and sleep (Barton and Feinberg, 2008). They teach physiology, functional anatomy and performing arts medicine (Spahn et al., 2001; Hildebrandt and Nubling, 2004; Zander et al., 2010), ergonomics (Martín López and Farías Martónez, 2013), injury prevention (Barton and Feinberg, 2008), and hearing health (Laursen and Chesky, 2014). They advocate concentration, mental rehearsal and imagery for effective practice and enhanced performance (Clark and Williamon, 2011). They recommend techniques such as visualization, breathing, relaxation, cognitive restructuring, and self-talk for performance anxiety (Barton and Feinberg, 2008; Clark and Williamon, 2011; Osborne et al., 2014; Spahn et al., 2016). They use behavioral exposure in the form of public performance (Clark and Williamon, 2011; Spahn et al., 2016) and offer classes in Feldenkrais Method and Alexander Technique (Zander et al., 2010; Su et al., 2012).

The reports listed above provide little information as to how curricula were designed and whether formative methodologies such as literature reviewing, feedback from a range of stakeholders, pilot testing and subsequent adjustments to the design were made. The distinction between health promotion and health education is almost never made explicitly. This matters because the latter should be evaluated in terms of the extent to which students' awareness, perceived competency, perceived knowledge *of* and perceived responsibility *for* health risks increased as a result of taking the course; to date, only Laursen and Chesky (2014) have reported these outcomes in relation to changes to a brass methods curriculum.

### Health and wellbeing for musicians: course structure, content, delivery and assessment

The course was designed as the major component of a module entitled Artist Development 1, compulsory for all first-year students at a tertiary-level music conservatoire in the UK. The other components were recording and self-promotion. The module took place over the first and second terms of the academic year (September-March) and consisted of ten weekly 1-hour lectures delivered to the whole cohort (104 students) and eight weekly 1-hour seminars delivered to ten small groups of 10–15 students. Seven of the lectures and five of the seminars related, broadly, to health and wellbeing.

Lecture 1, *How to practice more effectively*, was delivered by the Head of the School of Strings. Topics included deliberate practice; listening back to self-recordings; appraising ideal performances and designing exercises for overcoming identified weaknesses.

Lecture 2, *How to rehearse more effectively*, was delivered by the Head of Chamber Music with live illustrations from a first-year piano trio. Topics included warming up and rehearsing as a group; overcoming technical difficulties and problems with rhythm, articulation, bowing and breathing; intonation in groups with and without piano; learning how to identify errors, and to give and receive constructive criticism; the use of recordings when developing interpretations; responding and listening to the music while playing; interacting with the audience.

Lecture 3, *Introduction to health and wellbeing*, was delivered by the first author. Topics included the findings of recent research on the prevalence and symptoms of, and risk factors for MPA, PRMDs and hearing loss; healthy lifestyles (e.g., nutrition and sleep) and health-promoting behaviors (e.g., physical activity and reducing sedentary behavior); behavior-change strategies focusing on the concept of life skills as defined by WHO (1998).

Lecture 4, *Life skills for musicians including behavior change techniques*, was also delivered by the first author and focused on both health and music-making. Life skill topics included time management, exposure (e.g., to healthy options or public performance) and restriction (i.e., intentionally reducing exposure). Behavior change techniques included goal setting and self-monitoring (Michie et al., 2009; Dombrowski et al., 2012; see also Samdal et al., 2017); planning; self-talk; grading tasks; cognitive reframing (Brooks, 2014); disputation as a solution for reducing the impact of negative thoughts, in (McLeod, 2015).

Lecture 5, *Anatomy and physiology for musicians*, was delivered by a specialist in performing arts medicine. Topics included sensorimotor integration particularly in relation to MPA.

Lecture 6, *Managing music performance anxiety*, was delivered by a music psychologist. Topics included prevalence; symptoms; causes; and the relationship between arousal and performance quality. Potential solutions were suggested in the form of a toolbox of evidence-based strategies including peak performance approaches.

Lecture 7, *Presentation skills*, was delivered by a senior member of the School of Vocal Studies. The session focused on public speaking and included information on physical (e.g., voice warm-ups) and mental preparation.

Ten groups of students (three groups of singers, three groups of string players, two groups of keyboard players and two groups of wind, brass and percussion players) each took part in five seminars that were intended to reflect the content of the seven lectures. The first author facilitated all ten seminars entitled *Life skills for musicians*; the performing arts medicine specialist facilitated all those entitled *General ergonomics: How do I improve my posture?* The remaining seminars were entitled *Injury prevention and management, including hearing protection; Preparation for performance;* and *Successful careers: Time management, finances, life on tour* and were facilitated by a range of tutors including the fourth author.

Students were required to submit a portfolio of assessments including a 1,000-word essay in response to both the following questions: (1) Looking back on the Health and Wellbeing component of Artist Development 1, what new information, useful for your own music-making, have you learned from one lecture or one workshop/seminar?; (2) How have you been able to put this information into practice when making music (e.g., practicing, rehearsing, performing or studying more generally)?

### Health and wellbeing for musician: course evaluation

A mixed-methods approach to evaluation was adopted: quantitative analyses of within-subject data gathered at baseline and post-intervention, and between-group data (intervention vs. controls); and qualitative, semi-structured interviews (Matei et al., 2017). The research was approved by the institution's research ethics committee. While the course was compulsory for all first-year students, informed consent to participate in the research was given by completion of the questionnaires, which took about 30 min. These were administered at the beginning of the first lecture of the course in September 2016 and at the end of the last lecture in February 2017, via Bristol Online Surveys and as hard copy. The control group consisted of students who had been in the first year of their undergraduate studies in 2015–2016 (i.e., the year before the course was introduced) and responded to the same questionnaire, slightly modified, in March and April 2018, when they were third-year students.

#### Measures

The full questionnaire can be seen in the Appendix in Supplementary Material. It includes items reflecting relevant demographic data; hearing; primary and secondary outcomes.

*Hearing and use of hearing protection* were measured using 12 items adapted from Laitinen and Poulsen (2008).

*Primary outcomes* were measured using 15 items adapted from Laursen and Chesky (2014): perceived knowledge of seven topics covered in the course; awareness of potential risks to health associated with music performance; knowledge of potential risks to hearing, health and safety; responsibility for self-education and prevention of ill-health; and competency to implement recommendations for healthy performance. In order to assess the value they attached to the topics covered in the course, respondents were asked to rate their perceived importance, since health-related perceived importance has been associated with a higher likelihood of engaging in health promoting behaviors (Wardle and Steptoe, 1991; Näslund and Fredrikson, 1993; Orji et al., 2012). All ratings were made using 11-point scales from 0 (none) to 10 (greatest possible) or equivalent.

*Secondary outcomes* were measured as follows:

a) *General health*, a single item of self-rated health status of the RAND Short Form 36 Health Survey (SF-36; Ware and Sherbourne, 1992; McDowell, 2006), measured on a scale from 1 (excellent) to 5 (poor). Responses are recoded as scores of 100, 75, 50, 20, and 0, with higher values suggesting better perceived health.
b) *Health-related quality of life* (HRQoL): four items from the 15D scale (Sintonen, 1994): (problems with) sleeping, depression, distress, and (lack of) vitality, measured on a scale from 1 (normal) to 5 (severe).
c) *Health-promoting behaviors:* the Health Promoting Lifestyle Profile II questionnaire (HPLP II: Walker and Hill-Polerecky, 1996), consisting of 52 items representing six sub-scales: health responsibility (HR), physical activity (PA), nutrition (NU), spiritual growth (SG), interpersonal relations (IR), and stress management (SM), measured on a scale from 1 (never) to 4 (routinely).
d) *Self-efficacy* (i.e., self-appraisal of one's capability to deal with a situation or solve a problem), which might facilitate both engagement in health-promoting behaviors and maintenance of healthy habits (Kreutz et al., 2008): the Self-Efficacy Scale (SES: Schwarzer and Jerusalem, 1995): ten items, measured on a scale from 1 (not at all) to 4 (exactly true).
e) *Emotional states* during the previous week: the Positive and Negative Affect Schedule (PANAS: Watson et al., 1988), a set of 20 adjectives describing positive (10) and negative (10) affective states, measured on scales from 1 (very slightly) to 5 (extremely). Kreutz et al. (2008) argue that positive emotions may stimulate engagement in health-promoting behaviors and thereby reinforce them.
f) *Perceived stress:* the Perceived Stress Scale (PSS-10: Cohen and Williamson, 1988) was found to be a reliable and valid tool in a study of college students (Roberti et al., 2006): ten items relating to stress levels in the previous month, measured on a scale from 0 (never) to 4 (very often). Ratings are added to produce a total, rather than a mean score. The total score can range from 0 to 40.
g) *Frequency and severity of PRMDs*: two items adapted from Ackermann and Driscoll (2010), measured on 11-point Likert scales, from 0 (never) to 10 (constantly), and from 0 (none) to 10 (most severe) respectively.
h) *Perceived exertion*, to evaluate the amount of physical effort respondents needed to complete their daily practice routines: the Borg Rating Scale (Borg, 1998), which ranges from 6 (no exertion at all) to 20 (maximal exertion).

The respondents' 1,000-word essays, written for the purposes of course assessment, also served as a source of data.

## Analyses

Quantitative data were analyzed using IBM SPSS 22. Descriptive and inferential statistics are presented. Statistical significance was considered at *p* = 0.05. Confidence intervals of 95% were used throughout. Missing data were handled using listwise deletion for the exploration of changes across time and between groups.

For within-subject analyses, Wilcoxon Signed-Rank tests for paired samples were run, because the assumptions for normality were not met. Effect sizes were calculated using the following formula: *r* = *Z/*√*N* (where *N* is the total number of cases, not participants). The paired-samples sign test was run when the assumption of symmetrical distribution was not met.

For between-group analyses, Mann-Whitney *U*-tests for independent samples were used, while effect sizes were calculated using the following formula: η^2^ = *Z*^2^*/(N*−1*)* (where *N* is the total number of participants). For normally distributed data, independent *t*-tests were conducted.

Respondents' essays were anonymised and their titles (or content, in the absence of titles) categorized using open (bottom-up) coding, according to course topics covered.

## Results

### Sample characteristics: attrition rate and demographics

#### Intervention group

Of a total of 104 first-year undergraduate students enrolled on the course, only 13 did not complete the baseline questionnaire (12.5%). Of the 91 students who did, 81 (90%) completed the same questionnaire post-intervention: an attrition rate of only 10%. The mean age of these 81 respondents (37 males, 41 females, 3 undisclosed sex) was 19 years (range 18–26, *SD* = 1.34). Twenty-nine (36.3%) were singers, 19 (23.8%) were string players, 17 (21.3%) were wind and brass players, 11 were pianists (13.8%), three were composers (3.8%), and one was a percussionist (1.3%). The mean number of years they had sung, or played their main instrument, was 9.4 (range 2–18, *SD* = 3.09). They reported carrying out a mean of 14.3 h of personal practice per week (range 0–84 h, *SD* = 11.08).

#### Control group

Thirty-three third-year undergraduate students (18 male, 14 female, and one who preferred not to disclose their sex) with a mean age of 22 (range 20–27, *SD* = 1.71) completed the questionnaire either online or as hard copy in March–April 2018. Fifteen were string players (46.9%), six were keyboard players (18.8%), six were wind and brass players (18.8%), three were singers (9.4%), and two were composers (6.3%). Information on main instrument was missing for one respondent. They had played their main instruments for a mean of 12 years (range 7–18, *SD* = 3.16).

### Hearing and use of hearing protection

For the purposes of comparing the intervention group with controls, data from all the students who completed to the questionnaire at baseline, including those who did not complete it post-intervention, are shown in Table 1 as numbers and percentages of respondents to each question.

**Table 1:** Hearing and use of hearing protection.

	**Intervention (T1 only) N (%)**	**Control N (%)**
**USE OF HEARING PROTECTION**		
*Do you use ear protection aids (ear plugs/noise-reducing headphones)…*		
…*while practizing alone* (89 Intervention, 33 Control)		
Never/Seldom	80 (89.9%)	30 (90.9%)
Sometimes/Often/Always	9 (10.1%)	3 (9.1%)
…*at rehearsals with other players* (89 I, 33 C)		
Never/Seldom	71 (79.8%)	23 (69.7%)
Sometimes/Often/Always	18 (20.2%)	10 (30.3%)
…*at performances (my own)* (89 I, 33 C)		
Never/Seldom	82 (92.1%)	29 (87.9%)
Sometimes/Often/Always	7 (7.9%)	4 (12.1%)
…*at other people's performances* (90 I, 33 C)		
Never/Seldom	73 (81.1%)	21 (63.6%)
Sometimes/Often/Always	17 (18.9%)	11 (36.4%)
*Use of hearing protectors* (39 I users, 21 C users)		
I got used to wearing them right away	22 (56.4%)	7 (33.3%)
It took me weeks/months/years to get used to them	6 (15.4%)	7 (33.3%)
I didn't get used to them, but I use them anyway	7 (17.9%)	4 (19.0%)
I didn't get used to them, so I stopped using them	4 (10.25%)	3 (14.3%)
I have never used them	51	11
*Type of ear protection* (37 I users, 20 C users)		
Single use soft ear-plugs	10 (27%)	4 (20%)
Reusable (more expensive) soft ear plugs	26 (70.3%)	14 (70%)
Personally tailored, custom-made ear plugs	1 (2.7%)	2 (10%)
*While using your ear plugs, did you encounter any of the following difficulties?a 56 I, 38 C)*		
The ear plugs hindered my own performance	9 (16.1%)	11 (33.3%)
The ear plugs decreased my ability to hear the other player	15 (26.8%)	15 (45.5%)
Ear plugs were uncomfortable	10 (17.9%)	4 (12.1%)
Ear plugs were difficult to put into ears	12 (21.4%)	4 (12.1%)
Ear plugs made me feel dizzy	2 (7.1%)	0
Ear plugs caused a pressure sensation in my ear	8 (14.3%)	4 (12.1%)
*If your instrument is suitable for playing with mute (muffler), how often do you use it on your instrument? (40 I, 18 C)*		
Never/Seldom	29 (72.5%)	14 (77.8%)
Often/Always	11 (17.5%)	4 (22.2%)
**HEARING ISSUES**		
*Do you have tinnitus?* (88 I, 33 C) YES	7 (7.95%)	7 (21.2%)
*Do you experience hyperacusis?* (86 I, 33 C) YES	5 (5.8%)	5 (15.15%)
*Do you experience distortion?* (80 I, 33 C) YES	1 (1.25%)	0
*Do you experience diplacusis?* (80 I, 33 C) YES	0	0
*When was your hearing last checked?* (66 I, 32 C)	
I don't know	19 (28.8%)	4 (12.5%)
I have never had a hearing test	18 (27.3%)	10 (31.3%)
Over 10 years ago	5 (7.6%)	3 (9.4%)
6–10 years ago	9 (13.6%)	2 (6.3%)
4–5 years ago	3 (4.5%)	3 (9.4%)
1–3 years ago	6 (9.1%)	3 (9.4%)
In the last 12 months	6 (9.1%)	7 (21.9%)
*When your hearing was checked, were you told that you have hearing loss?* (40 I, 26 C)	
Yes	4 (10%)	0
No	29 (72.5%)	19 (73.1%)
Cannot say	7 (17.5%)	7 (26.9%)

*^a^Respondents could choose more than one option*.

#### Use of hearing protection

In both groups, minorities of respondents reported using hearing protection “sometimes,” “often” or “always” while practicing alone (10% of the intervention group and 9% of controls), and during their own performances (8% of the intervention group and 12% of controls). By contrast, 20% of the intervention group and 30% of controls reported using hearing protection while rehearsing with other people, and 19% of the intervention group and 36% of controls used it while listening to other people's performances. Seventy percent of those who did use hearing protection used reusable soft ear plugs. Of those whose instruments can be muted, 17.5% of the intervention group and 22.2% of controls reported using the mute “often” or “always.”

#### Experiences of using hearing protection

Fifty-six percent of the respondents in the intervention group who used hearing protection, but only 33% of controls, reported having got used to wearing them right away; another 33% of controls said it had taken them “weeks/months/years” to get used to them.

#### Difficulties using hearing protection

The most frequently-reported problems experienced by respondents in the intervention and control groups were a decrease in their ability to hear other players (26.8 and 45.5% respectively). The next most frequently-reported problems were difficulty inserting ear plugs (21.4% of intervention group responses) and hindrance to the player's own performance (33.3% of control group responses). The questionnaire included an invitation to report other problems: responses included “not being able to hear details in the sound;” “made listening to my sound more difficult;” “can't sing with them in;” “I felt isolated and anxious over the sounds I was making and tuning;” “I can hear my mouth moving—very distracting;” and “hear myself from within my mouth when playing”.

#### Hearing issues

Tinnitus was reported by 8% of the intervention group and 21% of controls, and hyperacusis by 6% and 22% respectively. Only one member of the intervention group experienced distortion and no-one reported diplacusis.

#### Hearing loss

While only 36% of the intervention group and 47% of controls had had a hearing test in the previous 10 years, only 10% of the former and none of the latter had been diagnosed with hearing loss.

### Primary outcomes

Descriptive and inferential statistics are shown in Table 2 for perceived knowledge and importance of topics covered in the course, and awareness and knowledge of potential risks to health.

**Table 2:** Perceived knowledge and importance of topics, awareness and knowledge of potential risks.

	***N***	***T1 (mean, SD)***	***N***	***T2 (mean, SD)***	***Z value***	***p***	***Effect size (r)***	***N***	***Control (mean, SD)***	***Z value (comparison with intervention group at T2)***	***p***	***Effect size (η^2^)***
**PERCEIVED KNOWLEDGE OF:**												
1.Effective practizing strategies	80	5.62 (2.02)	81	6.92 (1.60)	−4.32	<**0.001**	−**0.37**	33	7.18 (1.77)	−1.32	0.18	
2.Effective rehearsing strategies	80	5.21 (2.26)	79	6.40 (1.72)	−3.84	<**0.001**	−**0.32**	33	6.57 (2.00)	−1.04	0.29	
3.Learning and memorizing strategies+	80	5.76 (2.24)	81	6.39 (2.07)	−2.37	**0.01**	−**0.18**	33	6.51 (2.37)	−0.56	0.57	
4.Ergonomics/posture	80	5.56 (2.34)	81	6.19 (2.15)	−2.45	**0.01**	−**0.21**	33	6.42 (2.07)	−0.86	0.38	
5.Managing music performance anxiety	80	4.47 (2.58)	81	6.86 (1.85)	−4.97	<**0.001**	−**0.42**	32	5.93 (2.44)	−1.69	0.09	
6.Life skills and behavior change techniques+	80	4.87 (2.67)	81	6.39 (2.05)	−3.12	**0.002**	−**0.24**	33	5.42 (2.54)	−1.54	0.12	
7.Presentation skills+	79	5.64 (2.35)	81	6.28 (2.29)	−2.31	**0.02**	−**0.19**	33	6.45 (2.29)	−0.24	0.80	
**PERCEIVED IMPORTANCE OF:**												
1.Effective practizing strategies+	77	8.74 (1.49)	80	8.76 (1.72)	−0.15	0.87		32	8.62 (2.05)	−0.48	0.62	
2.Effective rehearsing strategies	77	8.63 (1.47)	80	8.48 (1.85)	−1.11	0.26		32	8.28 (2.06)	−0.62	0.52	
3. Learning and memorizing strategies	77	7.97 (1.85)	80	8.21 (1.79)	−1.30	0.19		32	7.68 (1.59)	−2.07	**0.03**	0.04
4.Ergonomics/posture+	77	8.36 (1.60)	80	8.33 (1.87)	0	1.00		32	7.78 (1.87)	−1.80	0.07	
5.Managing music performance anxiety+	76	8.42 (2.06)	80	8.17 (1.68)	−1.71	0.08		32	8.18 (2.62)	−0.86	0.38	
6.Life skills and behavior change techniques	77	7.64 (2.28)	80	7.61 (2.25)	−0.34	0.73		32	7.21 (2.63)	−0.99	0.31	
7.Presentation skills+	75	8.06 (1.83)	80	7.48 (2.27)	−0.91	0.35		32	7.31 (2.62)	−0.32	0.74	
**AWARENESS OF POTENTIAL RISKS**												
As a future professional musician, are you aware of any performance factors that are related to musculoskeletal injuries associated with learning and playing an instrument/singing?	80	5.36 (2.86)	80	6.62 (2.34)	−3.09	**0.002**	−**0.26**	33	6.87 (2.67)	−1.19	0.23	
Learning and performing music may involve hazards that have a negative impact on health.	81	5.77 (3.22)	81	6.03 (3.12)	−0.90	0.36		33	6.63 (3.21)	−1.20	0.22	
The way an individual plays a musical instrument/sings influences his/her level of risk of injury or health problems.	81	7.20 (2.57)	79	7.31 (2.60)	−0.32	0.74		33	8.06 (2.27)	−1.75	0.07	
**KNOWLEDGE OF POTENTIAL RISKS**												
Do you know what sound intensity levels are associated with hearing loss?	80	5.50 (2.98)	79	6.22 (2.78)	−2.09	**0.03**	−**0.17**	33	5.03 (3.82)	−1.83	0.06	
As a future professional musician, do you feel you have the resources, understanding, and knowledge to deal with the health and safety issues associated with learning and performing music?+	81	5.50 (2.30)	80	7.15 (1.92)	−5.03	<**0.001**	−**0.39**	33	6.51 (1.97)	−1.60	0.10	

+ *Results based on the Sign Test analysis (within-subjects)*.

#### Perceived knowledge

There were statistically significant increases from baseline to post-intervention in mean ratings for perceived knowledge of all topics covered in the course: effective practicing strategies (*Z* = −4.32, *p* < 0.001); effective rehearsing strategies (*Z* = −3.84, *p* < 0.001); learning and memorizing strategies (*Z* = −2.37, *p* = 0.01); ergonomics and posture (*Z* = −2.45, *p* < 0.01); managing MPA (*Z* = −4.97, *p* < 0.001); life skills and behavior change techniques (*Z* = −3.12, *p* = 0.002); presentation skills (*Z* = −2.31, *p* = 0.02). Small to medium effect sizes associated with these changes varied between *r* = 0.18 and *r* = 0.42 (Cohen, 1988). There was a trend such that respondents rated their perceived knowledge, post-intervention, higher than controls on managing MPA (*Z* = −1.69, *p* = 0.09) but the difference between means did not reach significance.

#### Perceived importance

Respondents rated their knowledge of effective learning and memorizing strategies, post-intervention, higher than controls (*Z* = −2.07, *p* = 0.03, η^2^ = 0.04), and tended to give higher ratings for the perceived importance of ergonomics and posture (*Z* = −1.80, *p* = 0.07) although the difference between means did not reach significance. Otherwise, there were no differences between the ratings of the intervention and control groups, nor changes from baseline to post-intervention.

#### Awareness of potential risks

There was a significant increase from baseline to post-intervention in ratings for one of the three items: awareness of performance factors related to musculoskeletal injuries associated with learning and playing an instrument/singing (*Z* = −3.09, *p* = 0.002, *r* = 0.26). There were no significant differences between the ratings of respondents, post-intervention, and controls.

#### Knowledge of potential risks

There were significant increases from baseline to post-intervention in ratings for both items: knowledge of sound intensity levels associated with hearing loss (*Z* = −2.09, *p* = 0.03, *r* = 0.17) and how to deal with the health and safety issues associated with learning and playing a musical instrument (*Z* = −5.03, *p* < 0.001, *r* = 0.39). There were no significant differences between the ratings of respondents, post-intervention, and controls. There was, however, a trend such that the former rated their knowledge of sound intensity levels higher than the latter (*Z* = −1.83, *p* = 0.06), although the difference between means did not reach significance.

#### Other primary outcomes

As shown in Table 3, there were no significant increases from baseline to post-intervention in ratings for responsibility for self-education and prevention of ill-health, or competence to implement recommendations for healthy performance. Nor, for these outcomes, were there any significant differences between the ratings of respondents, post-intervention, and controls.

**Table 3:** Perceived responsibility and competence.

	***N***	***T1(mean, SD)***	***N***	***T2(mean, SD)***	***Z value***	***p***	***N***	***Control(mean, SD)***	***Z value(comparison with intervention group at T2)***	***p***
**RESPONSIBILITY FOR SELF-EDUCATION AND PREVENTION OF ILL-HEALTH**										
As a future professional musician, do you feel responsible for being informed and educated about health and safety issues related to learning and performing music?+	81	7.38 (2.11)	80	7.63 (1.89)	−0.90	0.36	33	7.15 (1.97)	−1.16	0.24
Do you feel personally responsible for preventing health problems that may occur?	81	7.81 (1.78)	80	8.05 (1.82)	−0.71	0.47	33	7.51 (1.88)	−1.27	0.20
**COMPETENCE TO IMPLEMENT RECOMMENDATIONS FOR HEALTHY PERFORMANCE**										
As a future professional musician, are you prepared to address the current recommendations launched by relevant international organizations to aid in the prevention of health and safety concerns that may arise through the learning and performance of musical instruments/singing?+	81	7.92 (1.94)	80	7.53 (2.00)	−1.65	0.09	33	7.03 (1.97)	−1.04	0.29

+ *Results based on the Sign Test analysis (within-subjects)*.

### Secondary outcomes

Descriptive and inferential statistics are shown in Table 4.

**Table 4:** Secondary outcomes.

	***N***	***T1 (mean, SD)***	***N***	***T2 (mean, SD)***	***Z value***	***p***	**Effect size *(r)***	***N***	**Control *(mean, SD)***	***Z value (comparison with T2)***	***p***	**Effect size *(η^2^)***
**General health**	80	67.81 (18.73)	81	66.04 (19.48)	−0.81	0.41		33	59.84 (22.48)	−1.53	0.12	
**HRQoL**												
Sleeping	81	1.69 (0.70)	81	1.91 (0.77)	−2.77	**0.005**	−**0.21**	33	2.09 (0.91)	−0.96	0.33	
Depression	81	1.53 (0.76)	81	1.60 (0.83)	−1.13	0.25		33	2.36 (1.08)	−3.58	<**0.001**	0.11
Distress	81	1.95 (0.86)	81	2.20 (0.95)	−2.63	**0.009**	−**0.20**	33	2.70 (1.18)	−2.18	**0.02**	0.04
Vitality	81	1.49 (0.63)	81	1.65 (0.79)	−2.02	**0.04**	−**0.15**	33	2.30 (0.95)	−3.49	<**0.001**	0.10
**HPLPII**												
Health Responsibility (HR)+	79	1.91 (0.53)	81	1.94 (0.56)	−0.12	0.90		32	2.01 (0.46)	−1.13	0.25	
Physical Activity (PA)	74	2.28 (0.62)	79	2.38 (0.64)	−1.12	0.25		32	2.28 (0.62)	−0.77	0.44	
Nutrition (NU)	80	2.57 (0.52)	78	2.63 (0.56)	−0.96	0.33		32	2.67 (0.50)	−0.22	0.82	
Spiritual Growth (SG)++	77	2.90 (0.54)	79	2.87 (0.60)	−0.07	0.93		33	2.70 (0.54)	1.37	0.17	
Interpersonal Relations (IR)	78	3.06 (0.48)	79	3.04 (0.50)	−0.32	0.74		30	2.92 (0.54)	−1.37	0.17	
Stress Management (SM)++	80	2.33 (0.43)	80	2.42 (0.51)	−1.25	0.21		33	2.33 (0.41)	0.91	0.36	
**Self-efficacy**	79	3.00 (0.41)	80	3.09 (0.48)	−2.52	**0.01**	−**0.20**	33	3.13 (0.45)	−0.71	0.47	
**PANAS**												
Positive Affect (PA)	76	3.89 (0.65)	80	3.56 (0.70)	−4.02	<**0.001**	−**0.32**	32	3.21 (0.83)	−2.30	**0.02**	0.04
Negative Affect (NA)	76	1.77 (0.59)	81	1.94 (0.72)	−1.64	0.09		32	2.33 (0.72)	−2.68	<**0.01**	0.06
**Perceived stress**	81	17.19 (6.49)	77	17.85 (7.20)	−0.50	0.57		29	21.10 (5.32)	−2.28	<**0.02**	0.04
**PRMDs**												
How often do you suffer from a PRMD?	80	1.42 (2.09)	81	1.66 (2.43)	−0.64	0.51		32	2.56 (2.68)	−1.76	0.77	
Average severity of PRMD	80	1.30 (1.97)	80	1.55 (2.03)	−1.12	0.26		32	2.43 (2.44)	−1.78	0.75	
**Perceived exertion**	78	7.32 (2.37)	80	6.40 (2.51)	−3.05	**0.002**	−**0.24**	32	8.16 (2.71)	−3.22	**0.001**	0.09

+ Results based on the Sign Test analysis (within-group).

++ *Results based on independent t-tests (between-group)*.

#### General health

Means at both baseline and post-intervention were comparable to those obtained previously among musicians, but much lower than values among university students in the UK (Araujo et al., 2017). There were no significant mean differences from baseline to post-intervention, nor between intervention group and controls.

#### HRQoL

While means at baseline and post-intervention were low, there were nevertheless significant increases in ratings representing sleep problems (*Z* = −2.77, *p* = 0.005, *r* = 0.21), distress (*Z* = −2.63, *p* = 0.009, *r* = 0.20), and lack of vitality (*Z* = −2.02, *p* = 0.04, *r* = 0.15). In comparison with respondents post-intervention, controls experienced more severe depression (*Z* = −3.58, *p* < 0.001, η^2^ = 0.11), distress (*Z* = −2.18, *p* = 0.02, η^2^ = 0.04), and lack of vitality (*Z* = −3.49, *p* < 0.001, η^2^ = 0.10).

#### Health-promoting behaviors

The HPLPII showed acceptable to good internal reliability for the whole scale (Cronbach's alpha = 0.77) and subscales at T1 with the following alphas: HR = 0.83; PA = 0.81; NU = 0.73; SG = 0.84; IR = 0.78; SM = 0.67) and at T2 for the entire scale (alpha = 0.79) and subscales: HR = 0.81; PA = 0.80; NU = 0.75; SG = 0.87; IR = 0.82; SM = 0.72). The grand mean of all scores on HPLPII was 2.53 (SD = 0.36), indicating that respondents reported engaging in health-promoting behaviors “sometimes” or “often” (Kreutz et al., 2008; Panebianco-Warrens et al., 2015; Araujo et al., 2017). Means for the subscales representing health responsibility, physical activity and stress management were lower, and means for the subscales representing nutrition, spiritual growth and interpersonal relations were higher than the grand mean. There were no significant differences in ratings at baseline and post-intervention, nor between those of respondents, post-intervention, and controls (see Table 4 for mean ratings and standard deviations).

#### Self-efficacy

The SES scale showed good internal reliabilities at T1 and T2 (Cronbach's alphas = 0.86 and 0.89 respectively). Ratings increased significantly from baseline to post-intervention (*Z* = −2.52, *p* < 0.01, *r* = 0.20), although the grand mean at baseline was only 3.0 (*SD* = 0.41), lower than found in previous research in the UK (*M* = 3.57; *SD* = 0.63: Kreutz et al., 2008) and South Africa (*M* = 3.89; *SD* = 0.59: Panebianco-Warrens et al., 2015). There were no significant differences between the ratings of respondents, post-intervention, and controls.

### Emotional states

The PANAS scale showed good internal reliabilities at T1 (PA Cronbach's alpha = 0.87; NA = 0.83) and T2 (PA = 0.90; NA = 0.88). Ratings for positive affect decreased significantly from baseline to post-intervention (*Z* = −4.02, *p* < 0.001, *r* = 0.32), although the mean at baseline was 3.89 (*SD* = 0.65), higher than those reported by Kreutz et al. (2008) and Panebianco-Warrens et al. (2015), which were 3.43 (*SD* = 0.75) and 3.51 (*SD* = 0.74) respectively. There was a trend such that ratings for negative affect increased (*Z* = −1.64, *p* = 0.09), although significance was not reached; once again, the mean at baseline was 1.77 (*SD* = 0.59), lower than the means reported in the UK and South African research, which were 2.09 (*SD* = 0.73) and 2.40 (*SD* = 0.81) respectively. In comparison with respondents post-intervention, controls experienced lower positive affect (*Z* = −2.30, *p* = 0.02, η^2^ = 0.04) and higher negative affect (*Z* = −2.68, *p* < 01, η^2^ = 0.06).

#### Perceived stress

The PSS scale showed good internal reliability at T1 and T2 (Cronbach's alphas = 0.86 and 0.87 respectively). There was no significant difference between mean ratings at baseline and post-intervention, but in comparison with respondents, post-intervention, controls reported higher levels of stress (*Z* = −2.28, *p* < 0.02, η^2^ = 0.04).

#### PRMDs

There were no significant differences between mean ratings representing the frequency and severity of PRMDs at baseline and post-intervention, nor between the ratings of respondents, post-intervention, and controls. Both frequency and severity were comparatively low.

#### Perceived exertion

There was a significant decrease from baseline to post-intervention (*Z* = −3.05, *p* = 0.002, *r* = 0.24), although controls reported their daily practice routine to require more (albeit “very light”) effort (*Z* = −3.22, *p* < 0.001).

### Student assignments

A total of 103 essays was submitted. Just over half the students chose to write about managing MPA or life skills and behavior change techniques (see Table 5). Less popular topics included injury prevention (including hearing loss), vocal health, practice and memorization strategies and the psychophysical mechanisms of performance and Alexander Technique. Three students wrote about public speaking, and a small minority chose to discuss the health and wellbeing component of the module as a whole.

**Table 5:** Course topics covered in student assignments.

	***N* (%)**
Managing MPA	36 (34.9%)
Life skills and behavior change techniques	21 (20.3%)
Injury prevention (including hearing loss)	10 (9.7%)
Vocal health	9 (8.7%)
Practice and memorisation strategies	9 (8.7%)
The psychophysical mechanisms of performance and Alexander Technique	6 (5.8%)
Public speaking	3 (2.9%)
Variety of topics or about the module as a whole	9 (8.7%)
Total	103 (100%)

## Discussion

The present intervention study investigated the effects of a compulsory health education course on a range of health-related outcomes for undergraduate music students. The course covered not only physical and mental health, but also effective strategies for practicing, memorizing and rehearsing, and life skills and behavior-change tools inspired by health psychology. It is the first such course to be designed and evaluated at a British conservatoire. Within-subject data were gathered at the beginning and end of the intervention in September 2016 and February 2017, and control data were gathered for the purposes of the between-group analysis in March–April 2018.

### Hearing and use of hearing protection

Tinnitus and hyperacusis were reported by both groups of respondents, with a higher incidence in the (third-year) control group than in the (first-year) intervention group. Ten percent of the intervention group had been diagnosed with hearing loss, although minorities of respondents in both groups reported having had hearing tests in the previous 10 years.

Although respondents were more likely to use hearing protection when rehearsing with others and attending concerts, comparatively few members of either group used hearing protection, or, if appropriate, the mute on their instrument, while practicing alone. This could affect hearing, since private practice can cause over-exposure to risky levels of sound: O'Brien et al. (2013), for example, estimated that recommended limits may be reached after less than half the practice time reported by participants in their study.

The majority of users preferred reusable soft ear plugs to single-use soft plugs and the much more expensive custom-made versions; over half the intervention group users and a third of control group users reported getting used to them immediately while the remainder needed more time, persisted despite discomfort, or gave up using them. Typical problems with ear plugs included decreased ability to hear others, hindrance to own performance, difficulties with insertion and the sensation of pressure in the ear.

These findings support the results of recent research investigating the perceived advantages and disadvantages of using ear plugs, how they are used and musicians' strategies for wearing them (Beach and O'Brien, 2017). The authors of that study carried out in-depth interviews with 23 musicians in Australia and found that they felt comfortable using them, appreciated their discreetness, and enjoyed an enhanced clarity of sound. They did not find communicating with other people any more difficult, although they were concerned that other people might have a negative opinion of them. Perceived disadvantages included the occlusion effect, which could have contributed to their experience of reduced sound quality; they also reported an impaired ability to judge sound balance, intonation, tone, and timbre.

### Primary outcomes

Reassuringly, respondents reported increased knowledge of the topics covered in the course, including the sound intensity levels associated with hearing loss, and how to deal with the health and safety issues associated with learning and playing a musical instrument. They also reported increased awareness of performance factors related to potential musculoskeletal injuries. The ratings of students who had taken the course and those who had not did not differ significantly, perhaps because the control group had had informal exposure to the other topics covered in the course, with the possible exception of life skills and behavior change techniques, which were introduced in the context of an innovative lecture on preventative health, tools for the initiation and maintenance of healthy habits, and cognitive strategies for addressing thinking errors. Students who had taken the course also rated their ability to deal with relevant health and safety issues significantly higher than controls, but these issues are likely to have been reinforced throughout the period of the intervention by instrumental and vocal tutors and through health and safety briefings provided by the conservatoire.

The results support those of Laursen and Chesky (2014) in relation to their health education programme; their respondents also reported significantly increased knowledge post-intervention, and Laursen and Chesky argue that even minimal intervention can produce positive effects. While we asked respondents to rate the importance (i.e., the value) they attached to each of the topics covered in the course, we found only one significant difference between intervention and control groups: the latter attached less importance to effective learning and memorizing strategies than the former, perhaps because, as current third-year students, they were more confident in their ability to meet the demands being made on them to learn and memorize. There were no changes in perceived importance between baseline and post-intervention. This can be attributed to a ceiling effect: means ranged from 7.64 to 8.74 at baseline and from 7.48 to 8.76 at post-intervention, suggesting that students find these topics highly relevant to their studies.

### Secondary outcomes

The only desired secondary outcome to increase significantly from baseline to post-intervention was self-efficacy, which may or may not have been the result of the course. Other significant increases were in the wrong direction: sleep problems, distress and lack of vitality all increased significantly from baseline to post-intervention, and controls experienced more severe depression, distress and lack of vitality. Positive affect decreased significantly and there was a trend toward an increase in negative affect, while controls experienced lower positive and higher negative affect. Controls also reported higher levels of perceived stress.

We attribute these negative findings to the cumulative pressure on students over time. The first time the intervention group completed the questionnaire, they were in their second week at the conservatoire; post-intervention, they were facing deadlines for assignments to be submitted and recitals to be given. They may, however, have fared better than the control group simply by virtue of being a year younger. What we cannot know is the extent to which the health education course may have mitigated the demands perceived by the students in the intervention group. What we do know is that it might take longer for behavioral than cognitive changes to be made (Barton and Feinberg, 2008).

The mean ratings for reported perceived exertion (RPE) decreased significantly from baseline to post-intervention, and were higher for controls. Means were so low, representing “very very” to “very” light, although not surprisingly low, given that perceived exertion measures the level of physical effort and that scores for both frequency and severity of PRMDs were also low. For example, some exercise-based interventions have been associated with a positive impact on both PRMDs and RPE in the past (Ackermann et al., 2002; Kava et al., 2010; Chan et al., 2014a). However, the result is hard to interpret, given that perceived exertion may be influenced by both physiological and psychological factors (McCrary et al., 2016).

Finally, the categorization of student assignments to the topics covered in the course indicated those that respondents found of most interest or direct relevance to them, at this point in their studies: predominantly managing MPA and life skills and behavior change techniques.

The strengths of the study include the design and evaluation of the course, which was more rigorous than the majority of those reviewed above. First, the content of the course was based on a critical appraisal of the available literature on interventions to improve the health of musicians, theories from health psychology, and a clear conceptualization of health education, according to the WHO definition, as opposed to health promotion. Second, the course was compulsory. This partially explains the low attrition rate and reduces the likelihood of selection bias, although completion of questionnaires was not itself mandatory. The findings are therefore likely to be both more realistic and generalizable than evaluations of optional courses. Indeed, Spahn et al. (2001) suggest that compulsory courses may be more effective. Third, lectures were delivered and seminars facilitated by tutors who were all performing musicians, which may have helped to promote more intimate and informed interaction with students; in addition, three of the tutors specialized also in health psychology, performing arts medicine, and psychology respectively. Fourth, seminars were conducted in an informal, relaxed manner, enabling students to ask questions freely and tutors to tailor content to the needs of particular groups of students. Fifth, assignments were set in such a way as to bridge the gap between theory and practice: students were asked to reflect on what they had learned and how they implemented it in their music-making. Sixth, it was helpful to compare the experiences of the students who took the course with those of a control group (albeit a year older, with a further year's experience of conservatoire training), so as to contextualize learning within the broader context of undergraduate studies in music performance.

The limitations of the study must also be acknowledged. First, we would have increased the rigor of our approach by consulting a wider variety of health professionals when we designed the course, given its high level of interdisciplinarity. Second, the authors did not have the final say on the content of all the lectures and sessions delivered, other than those we delivered ourselves, and could not therefore monitor the extent to which the course as a whole was evidence-based. Our impression from reading the materials with which we were provided by tutors, and the students' essays, suggests that, on the whole, the evidence base was satisfactory. As a general point, we would nevertheless argue that justification is needed to include popular practices with little research evidence to support their use, such as the Alexander Technique (Klein et al., 2014; Baggoley, 2015; Aetna, 2016), in health education courses for musicians. Music students and their tutors have such full schedules (Clark and Williamon, 2011) that they should not be exposed to interventions unless there is evidence that they are likely to be effective. Third, the set of questionnaires we used was lengthy, and response fatigue might have affected students' responses. Fourth, we used measures of perceived rather than actual knowledge. Fifth, it was not possible, for ethical reasons, to recruit a control group of first-year students at the conservatoire who would have been deprived of taking the course, and because the course was deliberately designed to take over two terms it was not possible to deliver it twice, once in each term, so as to use a wait-list design. We should have recruited a control group of second-year students in 2016 but, due to changes of personnel at the institution, we could not obtain permission; it was not possible until 2017, by which time we had begun to report findings to colleagues, that we were able to run the course again (it is now part of the curriculum) and administer questionnaires to the control group. This solution does not, therefore, permit us to ascertain the extent to which differences between groups were pre-existing or the result of the control group's additional experience. Sixth, using assignments as a way of evaluating the course is potentially problematic in that students' choices of sessions to write about may have been guided less by interest in the topic or its relevance to them and more by the lecturer's clarity, communication and/or charisma, how informative the slides were and whether an easily-accessible list of references had been provided: such factors could have made certain topics more memorable or attractive for the purposes of fulfilling an assignment. Essay content may not have been entirely reliable, as students are likely to have been motivated by the wish to pass the course. Some students did not refer explicitly to the title of the relevant lecture or seminar/workshop, or the name of the tutor, so their essays had to be categorized on the basis of our knowledge of the content delivered; others referred to the course as a whole.

In the absence of a national curriculum for health, all institutions of higher education must develop their own approaches to health education, as do many university music departments and music conservatoires. The questions posed by Ralph Manchester in 2006 remain pertinent: “Who will develop this course? What topics will be included in the syllabus? Who will teach it? Will it be offered to freshmen or seniors, or can it be taken during any year? Can one course meet the needs of performance majors, music education majors, and others? Should we develop some minimal national requirements?” (Manchester, 2006, p. 95–96). Further questions could be asked, such as: When can a course be considered successful? What are its desired outcomes? How should they be measured? Once the content and delivery of a course have been evaluated, how should they be adjusted, if necessary? To what extent should students' requirements and feedback be taken into consideration, given the available evidence and the need, on occasion, to challenge their beliefs? Very few health courses have been formally evaluated to date, and reports of those that have been evaluated do not say how the course was improved as a result.

Although it has been argued for the last 25 years that health education for musicians should be evidence-based (Zaza, 1993) and one of the four HPSM recommendations endorses the use of a health promotion framework, the declarations and recommendations fail to mention the importance of evidence-based *teaching*. Indeed the first HPSM declaration includes the unsubstantiated claim that performance injuries are preventable (Manchester, 2006). There is now a wealth of research on musicians' playing-related health problems, and their management, but unless this is disseminated effectively to senior managers and educators, instrumental and vocal tutors, and students, there is a risk that conservatoires will maintain traditional practices rather than responding systematically to the best evidence available.

The topic of how music students, too, can be convinced that health education is a vital part of their training remains largely unexplored. Framing the objectives of health education courses as “performance-enhancing” rather than “preventative” is likely to be more attractive to students. After all, from the musician's perspective, performance quality is an outcome worth pursuing, and such a strategy could even be self-reinforcing, leading to greater self-efficacy and reduced anxiety (Kenny, 2005). It remains unclear, however, whether improved wellbeing necessarily leads to better performance (Osborne et al., 2014).

Researchers carrying out similar studies in future should consult the best available literature when designing courses and make more use of iterative processes. They should employ rigorous approaches to investigate the effectiveness of complex programmes, including exploring the acceptability of a course; piloting it; recruiting active control groups; and using a range of measures such as validated questionnaires, objective measures and qualitative data. They should conduct follow-up studies after longer periods, given that physical and psychological health are determined, at least in part, by health-promoting behaviors that individuals take time to establish (Barton and Feinberg, 2008; Zander et al., 2010). Finally, they should disseminate the findings of their evaluations to relevant stakeholders, examine, discuss, and ultimately implement the course as part of the curriculum, in the interests of providing high-quality evidence-based health education for music students.

Although the course described in the present study did not have the hoped-for impact on secondary outcomes including reported health-related behaviors, reduced PRMDs and stress, it was associated with improvements in primary outcomes relevant to health education, namely the perceived knowledge of topics covered in the course and awareness of health risks. Furthermore, the study itself is the first evaluation of a health education course for musicians that documents the process of designing the course on the basis of a rigorous assessment of the available evidence, and its incorporation in the “real world” context of a music conservatoire.

## Ethics statement

This study was carried out in accordance with the recommendations of the Conservatoires UK research ethics committee. The protocol was approved by the Conservatoires UK research ethics committee. All participants gave written informed consent in accordance with the Declaration of Helsinki.

## Data availability statement

The raw data supporting the conclusions of this manuscript will be made available by the authors, without undue reservation, to any qualified researcher.

## Author contributions

RM and JGi contributed to the design of the course, delivered some of the course sessions, and designed the study and procedures. RM collected the data, and analyzed the quantitative data. RM and JGi drafted the manuscript. All authors edited the manuscript, read and approved the submitted version.

### Conflict of interest statement

The authors declare that the research was conducted in the absence of any commercial or financial relationships that could be construed as a potential conflict of interest.

## Footnotes

^1^(Healthy Universities, 2017). www.healthyuniversities.ac.uk

^2^(Musical Impact, 2017). www.musicalimpact.org
