# Supplementary material for: Health Education for Musicians
Source: Front Psychol. 2018 Jul 16;9:1137. doi: 10.3389/fpsyg.2018.01137 (PMC6055059; doi:10.3389/fpsyg.2018.01137)
Supplement: Supplementary file 1 [file Data_Sheet_1.docx]

Appendix

**Health and Wellbeing Questionnaire**

Raluca Matei (PhD candidate) and Professor Jane Ginsborg (RNCM) are investigating music performance students’ health and wellbeing as part of the Better Practice work package of the AHRC-funded CUK-wide research project Musical Impact ([www.musicalimpact.org](http://www.musicalimpact.org)).

Please complete this questionnaire online and submit it at the end of the session, or complete it as legibly as possible and give it to Raluca at the end of the session. It should take you about 20 minutes.

If you have any questions while you are completing it, please ask Raluca, or you can contact her afterwards at [raluca.matei@student.rncm.ac.uk](mailto:raluca.matei@student.rncm.ac.uk), or Jane Ginsborg at [jane.ginsborg@rncm.ac.uk](mailto:jane.ginsborg@rncm.ac.uk).

By completing and submitting the questionnaire the researchers will assume that you have given your informed consent to take part in the research. You do not *have* to answer every question but it would be very helpful indeed for the research if you could do so.

Your name will be kept separately from your responses to the questionnaire. All the information that is collected about you will be kept strictly confidential. Any information about you that is disseminated will have your name removed so you cannot be identified by it.

The results of the research will be used to evaluate the effectiveness of the Health and Wellbeing component of the Artist Development 1 module and will be reported in Raluca’s PhD thesis and related outputs such as conference proceedings and journal articles.

Thank you for completing and submitting this questionnaire.

| **TODAY’S DATE :** |
| --- |

| 1. Questions about you |
| --- |
| 1. Are you (please tick): Male Female Prefer not to say |
| 2. How old are you (years / months)? _________________________________ |
| 3. Degree:  BMus (Hons) Classical Music 🗆 GRNCM (joint course students) 🗆 |
| 4. What is your main instrument? _____________________________________ |
| 6. Total number of years playing main instrument: _________________(years) |

1. **Please indicate which statements best describe your own state of health today by placing a tick in one box in each group below.**
2. **Sleeping**

I am able to sleep normally, i.e. I have no problems with sleeping. ❑

I have slight problems with sleeping, e.g. difficulty in falling

asleep, or sometimes waking at night. ❑

I have moderate problems with sleeping, e.g. disturbed sleep,

or feeling I have not slept enough. ❑

I have great problems with sleeping, e.g. having to use

sleeping pills often or routinely, or usually waking at night

and/or too early in the morning. ❑

I suffer severe sleeplessness, e.g. sleep is almost impossible

even with full use of sleeping pills, or I stay awake most of

the night. ❑

1. **Depression**

I do not feel at all sad, melancholic or depressed.❑

I feel slightly sad, melancholic or depressed. ❑

I feel moderately sad, melancholic or depressed. ❑

I feel very sad, melancholic or depressed. ❑

I feel extremely sad, melancholic or depressed. ❑

1. **Distress**

I do not feel at all anxious, stressed or nervous. ❑

I feel slightly anxious, stressed or nervous. ❑

I feel moderately anxious, stressed or nervous. ❑

I feel very anxious, stressed or nervous. ❑

I feel extremely anxious, stressed or nervous. ❑

1. **Vitality**

I feel healthy and energetic. ❑

I feel slightly weary, tired or feeble. ❑

I feel moderately weary, tired or feeble. ❑

I feel very weary, tired or feeble, almost exhausted. ❑

I feel extremely weary, tired or feeble, totally exhausted. ❑

1. **Your feelings and emotions. Please read each item and then put the number from the scale below next to each word to indicate the extent you have felt this way over the past week:**

| 1  Very slightly/  Not at All | 2  A Little | 3  Moderately | 4  Quite a Bit | 5  Extremely |
| --- | --- | --- | --- | --- |

| 1. Interested ___________ | 11. Irritable ____________ |
| --- | --- |
| 2. Distressed ___________ | 12. Alert ____________ |
| 3. Excited ___________ | 13. Ashamed ____________ |
| 4. Upset ___________ | 14. Inspired ____________ |
| 5. Strong ___________ | 15. Nervous ____________ |
| 6. Guilty ___________ | 16. Determined ____________ |
| 7. Scared ___________ | 17. Attentive ____________ |
| 8. Hostile ___________ | 18. Jittery ____________ |
| 9. Enthusiastic ___________ | 19. Active ____________ |
| 10. Proud ___________ | 20. Afraid ____________ |

1. **Your present way of life or personal habits. Please respond to each statement as accurately as possible, and try not to skip any item. Indicate the frequency with which you engage in each behaviour by circling**

**N** for never, **S** for sometimes, **O** for often, or **R** for routinely.

|  | Never | Sometimes | Often | Routinely |
| --- | --- | --- | --- | --- |
| 1.Discuss my problems and concerns with people close to me. | N | S | O | R |
| 2. Choose a diet low in fat, saturated fat, and cholesterol. | N | S | O | R |
| 3. Report any unusual signs or symptoms to a physician or other health professional. | N | S | O | R |
| 4. Follow a planned exercise programme. | N | S | O | R |
| 5. Get enough sleep. | N | S | O | R |
| 6. Feel I am growing and changing in positive ways. | N | S | O | R |
| 7. Praise other people easily for their achievements. | N | S | O | R |
| 8. Limit use of sugars and food containing sugar (sweets). | N | S | O | R |
| 9. Read or watch TV programmes about improving health. | N | S | O | R |
| 10. Exercise vigorously for 20 or more minutes at least three times a week (such as brisk walking, bicycling, aerobic dancing, using a stair climber). | N | S | O | R |
| 11. Take some time for relaxation each day. | N | S | O | R |
| 12. Believe that my life has purpose. | N | S | O | R |
| 13. Maintain meaningful and fulfilling relationships with others. | N | S | O | R |
| 14. Eat 3-5 servings of bread, cereal, rice and pasta each day. | N | S | O | R |
| 15. Question health professionals in order to understand their instructions. | N | S | O | R |
| 16. Take part in light to moderate physical activity (such as sustained walking 30-40 minutes 5 times or more times a week). | N | S | O | R |
| 17. Accept those things in my life which I can not change. | N | S | O | R |
| 18. Look forward to the future. | N | S | O | R |
| 19. Spend time with close friends. | N | S | O | R |
| 20. Eat 2-4 servings of fruit each day. | N | S | O | R |
| 21. Get a second opinion when I question my health care provider’s advice. | N | S | O | R |
| 22. Take part in leisure-time (recreational) physical activities (such as swimming, dancing, bicycling). | N | S | O | R |
| 23. Concentrate on pleasant thoughts at bedtime. | N | S | O | R |
| 24. Feel content and at peace with myself. | N | S | O | R |
| 25. Find it easy to show concern, love and warmth to others. | N | S | O | R |
| 26. Eat 3-5 servings of vegetables each day. | N | S | O | R |
| 27. Discuss my health concerns with health professionals. | N | S | O | R |
| 28. Do stretching exercises at least 3 times per week. | N | S | O | R |
| 29. Use specific methods to control my stress. | N | S | O | R |
| 30. Work toward long-term goals in my life. | N | S | O | R |
| 31. Touch and am touched by people I care about. | N | S | O | R |
| 32. Eat 2-3 servings of milk, yoghurt or cheese each day. | N | S | O | R |
| 33. Inspect my body at least monthly for physical changes/danger signs. | N | S | O | R |
| 34. Get exercise during usual daily activities (such as walking during lunch, using stairs instead of elevators, parking car away from destination and walking). | N | S | O | R |
| 35. Balance time between work and play. | N | S | O | R |
| 36. Find each day interesting and challenging. | N | S | O | R |
| 37. Find ways to meet my needs for intimacy. | N | S | O | R |
| 38. Eat only 2-3 servings from the meat, poultry, fish, dried beans, eggs, and nuts group each day. | N | S | O | R |
| 39. Ask for information from health professional about how to take good care of myself. | N | S | O | R |
| 40. Check my pulse rate when exercising. | N | S | O | R |
| 41. Practice relaxation or meditation for 15-20 minutes daily. | N | S | O | R |
| 42. Am aware of what is important to me in life. | N | S | O | R |
| 43. Get support from a network of caring people. | N | S | O | R |
| 44. Read labels to identify nutrients, fats, and sodium content in packaged food. | N | S | O | R |
| 45. Attend educational programs on personal health care. | N | S | O | R |
| 46. Reach my target heart rate when exercising. | N | S | O | R |
| 47. Pace myself to prevent tiredness. | N | S | O | R |
| 48. Feel connected with some force greater than myself. | N | S | O | R |
| 49. Settle conflicts with others through discussion and compromise. | N | S | O | R |
| 50. Eat breakfast. | N | S | O | R |
| 51. Seek guidance or counselling when necessary. | N | S | O | R |
| 52. Expose myself to new experiences and challenges. | N | S | O | R |

1. **Your feelings and thoughts during the last month. Please put a tick in the appropriate column to indicate how often you felt or thought a certain way.**

| 0 = Never | 1 = Almost never | 2 = Sometimes | 3 = Fairly Often | 4 = Very Often |
| --- | --- | --- | --- | --- |

|  | 0 | 1 | 2 | 3 | 4 |
| --- | --- | --- | --- | --- | --- |
| 1.In the last month, how often have you been upset because of something that happened unexpectedly? |  |  |  |  |  |
| 2. In the last month, how often have you felt that you were unable to control the important things in your life? |  |  |  |  |  |
| 3. In the last month, how often have you felt nervous and „stressed”? |  |  |  |  |  |
| 4. In the last month, how often have you felt confident about your ability to handle your personal problems? |  |  |  |  |  |
| 5. In the last month, how often have you felt that things were going your way? |  |  |  |  |  |
| 6. In the last month, how often have you found that you could not cope with all the things that you had to do? |  |  |  |  |  |
| 7. In the last month, how often have you been able to control irritations in your life? |  |  |  |  |  |
| 8. In the last month, how often have you felt that you were on top of things? |  |  |  |  |  |
| 9. In the last month, how often have you been angered because of things that were outside of your control? |  |  |  |  |  |
| 10. In the last month, how often have you felt difficulties were piling up so high that you could not overcome them? |  |  |  |  |  |

1. **Below are ten statements about yourself. Please indicate the extent to which each one is true of you by putting a tick in the appropriate column.**

|  | 1  Not at all true | 2  Hardly true | 3  Moderately true | 4  Exactly true |
| --- | --- | --- | --- | --- |
| 1.I can always manage to solve difficult problems if I try hard enough. |  |  |  |  |
| 2. If someone opposes me, I can find the means and ways to get what I want. |  |  |  |  |
| 3. It is easy for me to stick to my aims and accomplish my goals. |  |  |  |  |
| 4. I am confident that I could deal efficiently with unexpected events. |  |  |  |  |
| 5. Thanks to my resourcefulness, I know how to handle unforeseen situations. |  |  |  |  |
| 6. I can solve most problems if I invest the necessary effort. |  |  |  |  |
| 7. I can remain calm when facing difficulties because I can rely on my coping abilities. |  |  |  |  |
| 8. When I am confronted with a problem, I can usually find several solutions. |  |  |  |  |
| 9. If I am in trouble, I can usually think of a solution. |  |  |  |  |
| 10. I can usually handle whatever comes my way. |  |  |  |  |

1. **Performance related musculoskeletal disorders (PRMDs) may be defined as any pain, weakness, numbness, tingling or any other symptoms that interfere with your ability to play your instrument at the level you are accustomed to. This definition does not include mild transient aches or pains.**

1. Please indicate how often you suffer from a PRMD by circling the most appropriate number:

Never 0 1 2 3 4 5 6 7 8 9 10 Constantly

2. Please indicate on the line below the average severity of any PRMD that you suffer from:

None 0 1 2 3 4 5 6 7 8 9 10 Most severe

1. **Please rate the amount of effort that you feel it takes you to complete an average daily practice routine. This is done by scoring your rating of Perceived Exertion (RPE). You are required to choose a number between 6 and 20 that corresponds to the physical exertion of performing this practice. For example; the number 6 represents an activity that requires no effort. The number 13 means that the exercise feels somewhat hard at the time, while the number 20 represents maximal effort.**

Please circle the number below that you feel best represents the degree of effort required to get through your daily hours of practice:

RPE SCALE

6

7 very, very light

8

9 very light

10

11 fairly light

12

13 somewhat hard

14

15 hard

16

17 very hard

18

19 very, very hard

20

1. **Your hearing and use of hearing protection**

1. Do you use ear protection aids (ear plugs/noise-reducing headphones) ?

|  | Never | Seldom | Sometimes | Often | Always |
| --- | --- | --- | --- | --- | --- |
| While practising alone |  |  |  |  |  |
| At rehearsals with other players |  |  |  |  |  |
| At performances (my own) |  |  |  |  |  |
| Other people’s performances |  |  |  |  |  |

2.Use of hearing protectors

I got used to wearing them right away ❑

It took me weeks/months/years (circle the correct choice) to get used to them ❑ I didn’t get used to them, but I use them anyway ❑

I didn’t get used to them, so I stopped using them ❑

I have never used them (please skip to Question 8 in this section) ❑

3. The type of ear protection aids I use

Single use soft ear-plugs ❑

Reusable (more expensive) soft ear-plugs ❑

Personally tailored, custom-made ear plugs❑

4. While using your ear plugs, did you encounter any of the following difficulties?

The ear plugs hindered my own performance ❑

The ear plugs decreased my ability to hear the other player ❑

Ear plugs were uncomfortable ❑

Ear plugs were difficult to put into ears ❑

Ear plugs caused me an ear infection ❑

Ear plugs made me feel dizzy ❑

Ear plugs caused a pressure sensation in my ear ❑

If other, what? ...........................................

5.Is your instrument suitable for playing with mute (muffler)? (please circle)

Yes No

6.If yes, how often do you use it on your instrument? (please circle)

Never Seldom Often Always

**Tinnitus** = *a sound of duration of minimum 5 minutes, an occasional sensation of a ringing, roaring, or buzzing sound in the ears or head even though no such sound is present*

7. Do you have tinnitus?

**Hyperacusis** (high sensitivity to sound) = *abnormal sensitivity to everyday sound levels or noises. Often there is also sensitivity to high pitched sounds*.

8. Do you experience hyperacusis?

❑ yes, since _____________ ❑ no

**Distortion** = *when sound reaches a certain level, it is perceived as being impure, cracked, distorted*

9. Do you experience distortion?

❑ yes, since [date]______________ ❑ no

**Diplacusis** = *the pitch of a sound presented to both ears is heard differently in each of the two ears*

10. Do you experience diplacusis?

❑ yes, since [date]______, ❑ no

11. When was your hearing last checked?

In the last 12 months ❑

1-3 years ago ❑

4-5 years ago ❑

6-10 years ago ❑

Over 10 years ago ❑

I don’t know ❑

I have never had a hearing test ❑

12. When your hearing was checked, were you told that you have hearing loss? (Please circle)

Yes No Cannot say

1. **Your responsibility, awareness, knowledge, competency and attitude as a future professional musician with respect to health and wellbeing**

1.Please rate your current level of knowledge of the following topics (which you will be exploring as part of the AD1 course) as applied to music making by circling the appropriate number (in all cases 0=none and 10=greatest possible).

| *Topic* |  |
| --- | --- |
| 1. Effective practising strategies | 0 1 2 3 4 5 6 7 8 9 10 |
| 2. Effective rehearsing strategies | 0 1 2 3 4 5 6 7 8 9 10 |
| 3. Learning and memorising strategies | 0 1 2 3 4 5 6 7 8 9 10 |
| 4. Ergonomics/posture | 0 1 2 3 4 5 6 7 8 9 10 |
| 5. Music performance anxiety | 0 1 2 3 4 5 6 7 8 9 10 |
| 6. Life skills and behaviour change techniques | 0 1 2 3 4 5 6 7 8 9 10 |
| 7. Presentation skills | 0 1 2 3 4 5 6 7 8 9 10 |

2. How important do you regard the following topics (which you will be exploring as part of the AD1 course) in relation to how well you perform musically on your instrument? Please circle the appropriate number (in all cases 0=none and 10=greatest possible).

| *Topic* | *Effect of each topic on performance* |
| --- | --- |
| 1. Effective practising strategies | 0 1 2 3 4 5 6 7 8 9 10 |
| 2. Effective rehearsing strategies | 0 1 2 3 4 5 6 7 8 9 10 |
| 3. Learning and memorising strategies | 0 1 2 3 4 5 6 7 8 9 10 |
| 4. Ergonomics/posture | 0 1 2 3 4 5 6 7 8 9 10 |
| 5. Music performance anxiety | 0 1 2 3 4 5 6 7 8 9 10 |
| 6. Life skills and behaviour change techniques | 0 1 2 3 4 5 6 7 8 9 10 |
| 7. Presentation skills | 0 1 2 3 4 5 6 7 8 9 10 |

**Please respond to the statements below using the scale 0–10 (please circle).**

3. Learning and performing music may involve hazards that have a negative impact on health.

Do Not Agree 0 1 2 3 4 5 6 7 8 9 10 Totally Agree

4. The way an individual plays a musical instrument/sings influences his/her level of risk of injury or health problems.

Do Not Agree 0 1 2 3 4 5 6 7 8 9 10 Totally Agree

5. As a future professional musician, are you aware of any performance factors that are related to musculoskeletal injuries associated with learning and playing an instrument/singing?

Not at All 0 1 2 3 4 5 6 7 8 9 10 Completely

6. Do you know what sound intensity levels are associated with hearing loss?

Not at All 0 1 2 3 4 5 6 7 8 9 10 Completely

7. As a future professional musician, do you feel you have the resources, understanding, and knowledge to deal with the health and safety issues associated with learning and performing a musical instrument/singing?

Not at All 0 1 2 3 4 5 6 7 8 9 10 Completely

8. As a future professional musician, do you feel responsible for being informed and educated about health and safety issues related to learning and performing music?

Not at All 0 1 2 3 4 5 6 7 8 9 10 Completely

9. Do you feel personally responsible for preventing health problems that may occur?

Not at All 0 1 2 3 4 5 6 7 8 9 10 Completely

10.As a future professional musician, are you prepared to address the current recommendations launched by relevant international organisations to aid in the prevention of health and safety concerns that may arise through the learning and performance of musical instruments/singing?

Not at All 0 1 2 3 4 5 6 7 8 9 10 Completely

Thank you very much for completing this questionnaire!

Please give it to Raluca now. Alternatively, you can submit it online during the coming week or finish completing it in your own time, as long as you submit it to her next week.

If any issues have arisen for you as a result of completing this questionnaire, please contact one of the student counsellors, Bryan Fox and Claire Donoghue, via telephone

(0161 907 5324) or email [counselling@rncm.ac.uk](mailto:counselling@rncm.ac.uk).
